# Evaluation of Viremia Frequencies of a Novel Human Pegivirus by Using Bioinformatic Screening and PCR

**DOI:** 10.3201/eid2204.151812

**Published:** 2016-04

**Authors:** David Bonsall, William F. Gregory, Camilla L.C. Ip, Sharyne Donfield, James Iles, M. Azim Ansari, Paolo Piazza, Amy Trebes, Anthony Brown, John Frater, Oliver G. Pybus, Phillip Goulder, Paul Klenerman, Rory Bowden, Edward D. Gomperts, Eleanor Barnes, Amit Kapoor, Colin P. Sharp, Peter Simmonds

**Affiliations:** University of Oxford, Oxford, UK (D. Bonsall, C.L.C. Ip, J. Iles, M.A. Ansari, P. Piazza, A. Trebes, A. Brown, J. Frater, O.G. Pybus, P. Goulder, P. Klenerman, R. Bowden, E. Barnes, P. Simmonds);; University of Edinburgh Easter Bush, Edinburgh, Scotland, UK (W.F. Gregory, C.P. Sharp);; Rho, Inc., Chapel Hill, North Carolina, USA (S. Donfield);; The Childrens' Hospital, Los Angeles, California, USA (E.D. Gomperts);; The Research Institute at Nationwide Children's Hospital, Columbus, Ohio, USA (A. Kapoor)

**Keywords:** Pegivirus, Hepegivirus, Hepacivirus, Flaviviridae, HHpgV-1, HHpgV-1, HCV, viruses, persons who inject drugs, hemophilia, transfusion, parenteral, sexually transmitted disease, bloodborne pathogens, virus persistence, viruses

## Abstract

Next-generation sequencing has critical applications in virus discovery, diagnostics, and environmental surveillance. We used metagenomic sequence libraries for retrospective screening of plasma samples for the recently discovered human hepegivirus 1 (HHpgV-1). From a cohort of 150 hepatitis C virus (HCV)–positive case-patients, we identified 2 persons with HHpgV-1 viremia and a high frequency of human pegivirus (HPgV) viremia (14%). Detection of HHpgV-1 and HPgV was concordant with parallel PCR-based screening using conserved primers matching groups 1 (HPgV) and 2 (HHPgV-1) nonstructural 3 region sequences. PCR identified 1 HHPgV-1–positive person with viremia from a group of 195 persons with hemophilia who had been exposed to nonvirally inactivated factor VII/IX; 18 (9%) were HPgV-positive. Relative to HCV and HPgV, active infections with HHpgV-1 were infrequently detected in blood, even in groups that had substantial parenteral exposure. Our findings are consistent with lower transmissibility or higher rates of virus clearance for HHpgV-1 than for other bloodborne human flaviviruses.

## Abstract 2

Bioinformatic screening and PCR-based approaches detected active infection with human hepegivirus-1 in exposed populations.

The development of next-generation sequencing methods and related molecular tools has greatly increased the pace of virus discovery (*1*,*2*), and these methods have become widely used for the investigation of novel zoonotic infections. Examples in which next-generation sequencing methods have identified novel viral agents associated with disease outbreaks include severe fever with thrombocytopenia virus (SFTV) in China (*3*), a bunyavirus in the United States (*4*), and a novel rhabdovirus in Central Africa (*5*). Using such methods, 2 authors of this study (A.K. and P.S.) recently described a novel flavivirus, distantly related to human pegivirus (HPgV, formerly described as GB virus C or hepatitis G virus) but with several genome attributes, such as a type IV internal ribosomal entry site (IRES), possession of a core-like protein, and a heavily glycosylated envelope protein that show greater affinity with hepatitis C virus (HCV) and other members of the genus *Hepacivirus* (*6*). The virus, named human hepegivirus 1 (HHpgV-1) to reflect these mosaic characteristics, was detected in 2 blood recipients and as a persistent infection in 2 persons with hemophilia exposed previously to nonvirally inactivated factor VIII/IX concentrates.

Following the example of the previous use of metagenomic libraries to detect human pathogens (*7*–*9*), we developed a bioinformatics-based method to screen existing libraries for HHpgV-1 and HPgV sequences from previously tested persons in the United Kingdom, enabling viremia frequencies in different risk groups to be estimated. Samples used to generate libraries with and without HHpgV-1 sequences were retrieved and used to validate the specificity and sensitivity of a newly developed reverse transcription PCR (RT-PCR)–based method for sample screening. This method was subsequently used to screen samples from patients extensively treated with nonvirally inactivated factor VIII or IX concentrates and controls.

## Methods

### Samples

We obtained samples from 195 persons with hemophilia from the Hemophilia Growth and Development Study (HGDS) cohort (*10*). We obtained metagenomic datasets used for bioinformatic screening from OxBRC Prospective Cohort Study in Hepatitis C (ethics reference 09/H0604/20), the Short Pulse Antiretroviral Therapy at seroConversion cohort (*11*), Thames Valley HIV Cohort Study (*12*), and a cohort in the Democratic Republic of the Congo (DRC) (*13*). All datasets were derived by using nontargeted viral RNaseq from total plasma RNA and the Illumina Hiseq sequencing platform (Illumina Inc., San Diego, CA, USA).

### Screening Assays for Group 1 and Group 2 Pegiviruses

 RNA was extracted from 200 μL of pooled or 20 μL of individual plasma by using the RNeasy Kit (QIAGEN, Hilden, Germany) and recovered in 30 μL of nuclease-free water. First-strand cDNA was synthesized from 6 μL of recovered RNA by using Superscript III reverse transcription (Life Technologies, Carlsbad, CA, USA) with random hexamer primers. Nested PCRs were performed by using GoTaq DNA polymerase (Promega, Madison, WI, USA) and the primers described in Table 1. First round reactions were obtained by using 2 μL of cDNA as a template under these conditions: 40 cycles of 18 seconds at 94°C, 21 seconds at 50°C and 60 seconds at 72°C, and a final extension step of 5 minutes at 72°C. Second round reactions were done by using 2 μL of first round template under identical conditions.

**Table 1:** Primer sequences used for PCR screening of human pegivirus groups

Orientation	Position†	Sequence, 5′ → 3′
HHpgV-1/group 2 primers		
Sense, outer	4,488	CGTSGTSMTYTGYGACGAGTGCCA
Antisense, outer	5,021	CCRCGCCGCTGCATVCGSAAYGC
Sense, inner	4,723	CAYGYDATCTTYTGYCACTCGAAGG
Antisense, inner	4,900	CRAAGTTBCCDGTGTAGCCDGTGGA
HPgV/group 1 primers		
Orientation	Position‡	
Sense, outer	3,931	GSGCNATGGGNCCNTAYATGGA
Antisense, outer	4,546	GTNACYTCVACNACCTCCTCYACCA
Sense, inner	4,092	GTGGTNATHTGYGAYGAGTGYCA
Antisense, inner	4,357	TCRCACTCMRCCTTKGARTGRCARAA

*HHpgV, human hepegivirus; HPgV, human pegivirus.  †Position of 5′ base in the AK-790 genome (GenBank accession no. KT439329). ‡Position of 5′ base in the HPgV Iowa genome (GenBank accession no. AF121950).

### Metagenomic Sequencing and Bioinformatics

Metagenomic datasets used in this study had previously been sequenced on the Illumina platform from sequencing libraries synthesized with either the NEBNext mRNA Sample Prep Kit for Illumina (New England Biolabs, Ipswich, MA, USA), or the NEBNext Ultra Directional RNA Library Prep Kit for Illumina (New England Biolabs) with modifications to the manufacturer’s protocols (*10*). Datasets in .bam format were depleted of human reads by using the bowtie method to map them to the HG19 human genome and convert them to fasta files by using custom awk scripts, then were piped to the blastn program (BLAST+ version 2.2.25; http://blast.ncbi.nlm.nih.gov/Blast.cgi) by using a nucleotide database of all GenBank viral reference genomes and the initial HHpgV-1 variant, AK-790 (GenBank accession no. KT439329). All hits with E values <0.01 were accepted. Datasets containing HHPgV-1 or HPgV-1 sequence data were subjected to a custom-made assembly pipeline by which reads were trimmed of low PHRED quality bases (QUASR, Sourceforge, http://wwwsourceforge.net) and adaptor sequences, before virus reads (identified by using blastn) were assembled by using Vicuna (*14*) and VFAT software (http://www.broadinstitute.org/scientific-community/science/projects/viral-genomics/v-fat). 

RNA folding energies and ratios of non-synonymous to synonymous nucleotide substitutions (dN/dS) were calculated for consensus whole-genome sequences by using SSE version 1.2 (*15*). Complete genome sequences of HHpgV-1 and HPgV obtained in this study have been submitted to GenBank (accession nos. LT009476–LT009494).

## Results

### Library Screening in Silico

We used the assembled sequence of AK-790 as a reference to screen libraries of metagenomic sequence reads derived from plasma samples from 120 HCV-infected persons (primarily infected through injected drugs), 36 persons infected with HIV-1 from sexual contact, and 30 persons who were co-infected with HCV and HIV-1 (Table 2). From these samples, a total of 3 sequence libraries contained HHpgV-1 sequences. However, only 2 reads were detected in sample D1212, and these were identical in sequence to those of D1220. Because of the possibility of extraneous contamination of either the sample position in the sequencer or of the sequence dataset (e.g., through misidentified tags), we provisionally considered D1212 to be HHpgV-1 negative.

**Table 2:** Detection frequencies of HPgV by sequence library screening and PCR*

Diagnostic method	Diagnoses and characteristics	No. tested	No. (%) HHpgV-1+	No. (%) HPgV+
Bioinformatics				
	HIV+	36	0	3 (8.3)
	HIV+/HCV+/PWID	30	0	3 (10.0)
	HCV+/PWID	120	2 (1.7)	14 (11.6)
PCR				
	Hemophilia†	195	1 (0.5)	18 (9.2)
	Control group‡	50	0	1 (2.0)

*HPgV, human pegivirus; HHpgV, human hepegivirus; +, positive;HCV, hepatitis C virus; PWID, persons who inject drugs. †Previously exposed to virally inactivated factor VIII/IX; all seropositive for HCV. ‡No history of parenteral exposure; HIV-1 and HCV negative.

**Figure 1:**
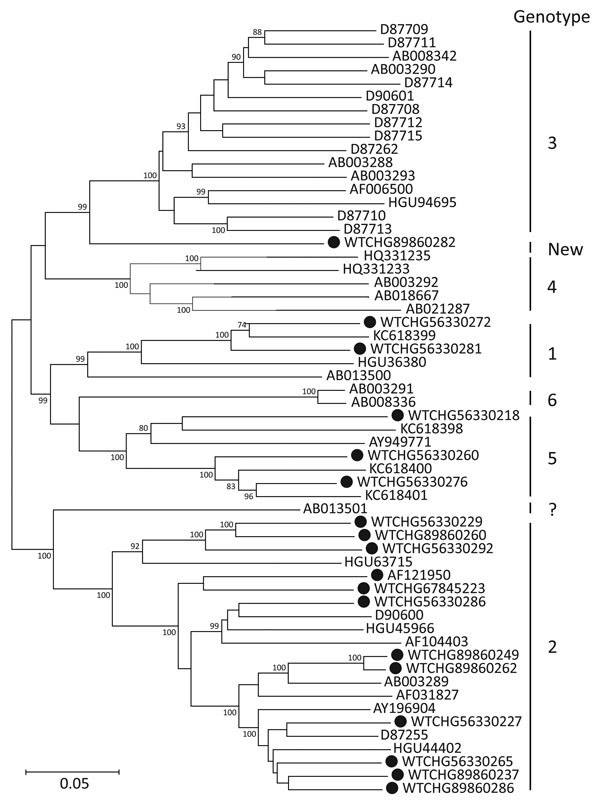
Maximum-likelihood phylogenetic analysis of complete genome sequences of human pegivirus assembled in this study (black circles) compared with available human hepegivirus (HPgV) sequences of genotypes 1–6 published in GenBank (accession numbers shown). The tree was constructed by using the maximum-likelihood algorithm implemented in the MEGA6 software package (*16*). For this dataset, the optimum maximum model was general time reversible model (*18*) with a gamma distribution (5 rates) and invariant sites. Phylogenetic analysis of each dataset used 100 bootstrap resamplings to infer the robustness of groupings. Genotypes previously assigned to HPgV sequences are shown on the right with the exception of sequence AB013501 (from the United Kingdom, shown with genotype “?”). Scale bar indicates nucleotide substitutions per site.

**Figure 2:**
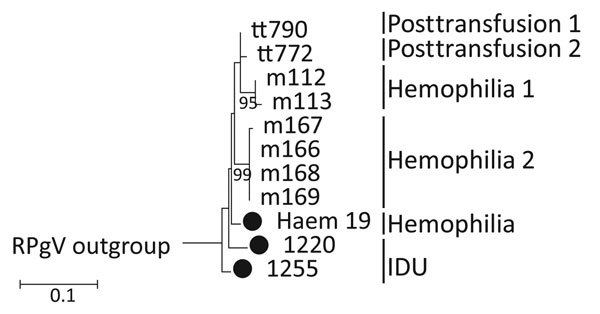
Maximum-likelihood (ML) phylogenetic analysis of human pegivirus sequences. NS3 region sequences (positions 4609–4880 as numbered in the AK-790 reference sequence, denoted here as tt790) were selected to overlap with sequences from PCR-derived amplicons generated in this study (black circles) and partial NS3 region sequences reported previously (*6*). The tree was constructed by using the maximum likelihood algorithm implemented in the MEGA6 software package (*16*). The optimum ML model (lowest Bayesian information criterion score and typically greatest ML value) was Kimura 2 parameter and invariant sites. Phylogenetic analysis of each dataset used 100 bootstrap re-samplings to infer the robustness of groupings. The tree was rooted with a rat pegivirus sequence (GenBank accession no. KC815311, not shown). IDU, injection drug use; NS, nonstructural. Scale bar indicates nucleotide substitutions per site.

By using the sequence reads, we obtained near-complete genome sequences of HHpgV-1 from the 2 positive samples (Table 2); their divergence, and other sequence characteristics were compared with those of the AK-790 prototype sequence (Figure 1; Table 3). Sequences were >99% complete with 5′ and 3′ ends approximately co-terminus with the HHpgV-1 prototype sequence (D1255 and D1220 lacked 12 and 23 bases at the 5′ end, respectively, and D1255 had a 23 base extension at the 3′ end). Sequences were ≈5% divergent from each other and from AK-790 over the length of the genome; most were at synonymous sites that left protein encoding unchanged (dN/dS 0.170–0.193). Sequences were also phylogenetically distinct from the larger dataset of variants sequenced in the nonstructural 3 (NS3) region (Figure 2; *6**,**16*). Genomes of both HHpgV-1 variants showed bioinformatic evidence for genome scale–ordered RNA structure (*17*), with mean folding energy differences in the coding region of 7.6% and 8.3% for samples D1255 and D1220, respectively, which is similar to that calculated for AK-790 (7.6%) but lower than that calculated for HPgV (Table 3) and other pegiviruses (*6*).

**Table 3:** Read depth and divergence of HPgV sequences obtained by using metagenomic screening*

Virus, ID no.	Case-patient origin	Type†	Length	Coverage, %	Reads	Div, %‡	dN/dS	MFED, %§
HHpgV-1, n = 2								
D1220	UK	NA	9,550	99.8	247,798	4.7	0.193	7.6
D1255	UK	NA	9,503	99.6	101,951	5.1	0.170	8.3
HPgV, n = 20								
89859249	UK	2	9,383	99.9	19,231,581	10.0	0.031	11.4
89859262	UK	2	9,388	99.8	6,766,497	9.9	0.029	12.4
56330218	South Africa	5	9,364	99.7	4,242,732	13.6	0.043	11.6
56330227	Australia	2	9,366	99.7	2,411,508	9.9	0.031	12.0
56330281	DRC	1	9,362	99.7	2,297,064	13.5	0.048	13.2
67845223	UK	2	9,382	99.9	1,332,130	9.4	0.023	11.5
56330260	UK	5	9,365	99.7	1,306,864	13.7	0.043	12.1
56330265	UK	2	9,425	99.7	1,178,508	9.5	0.035	12.5
56330292	NA	2	9,356	99.6	1,021,219	11.5	0.035	11.9
89860237	UK	2	9,367	99.7	953,683	9.5	0.035	11.0
56330272	DRC	1	9,340	99.4	707,495	13.6	0.052	12.0
56330229	UK	2	9,366	99.7	698,622	11.5	0.036	12.6
56330276	DRC	5	9,337	99.4	612,424	13.3	0.042	12.3
89860282	UK	Novel	8,443	89.9	574,652	13.2	0.049	10.5
89860286	UK	2	8,923	95.0	513,420	9.6	0.035	10.3
89860260	UK	2	8,983	95.6	512,515	11.6	0.041	10.5
56330286	NA	2	9,355	99.6	454,106	9.6	0.022	12.5
56330228	Australia	ND	436	4.6	68,351	8.6	0.019	ND
56330250	UK	ND	423	4.5	38,083	11.6	0.043	ND
89860212	UK	ND	1,117	11.9	17,226	8.6	0.068	ND
*HPgV, human pegivirus; ID, identification; Div, sequence divergence; dN/dS: ratio of nonsynonymous (dN) to synonymous (dS) substitutions; MFED, mean folding energy difference; HHpgV, human hepegivirus; UK, United Kingdom; NA, not applicable (no genotypes of HHpgV-1 are currently assigned); DRC, Democratic Republic of Congo; ND, not done (insufficient sequence length). †Genotype based on phylogenetic analysis of complete genome sequences (Figure 1). ‡Comparison with AK790 prototype sequence (HHPgV-1) or AF121950 (HPgV; genotype 2). §Difference in minimum folding energy of sequences compared with those of sequence order-randomized controls (MFED) (*16*).								

The 2 HHPgV-1 samples originated from HCV-infected men enrolled in the OxBRC Prospective Cohort Study in Hepatitis C; samples were collected before initiation of antiviral treatment with telaprevir, pegylated-interferon, and ribavirin. Sample D1255 was collected from a person with a history of injection drug use, 50 years of age, 12 weeks before successful eradication of HCV genotype 1b virus; at the time of the study, he had remained under observation for a segment IV liver lesion, cirrhosis, and raised alphafetoprotein. Sample D1220 was collected from a patient 58 years of age who had genotype 1a HCV infection and died from decompensated liver failure 4 weeks into treatment. Both patients denied receiving blood or donor blood–derived products at the time of enrollment, and neither patient received blood products before sampling, according to hospital medical records and local transfusion service records.

As a control for the bioinformatic screening method, the same samples were screened for human pegivirus (HPgV) by using 60 whole pegivirus genomes from the US National Center for Biotechnology Information nucleotide database (http://www.ncbi.nlm.nih.gov/nuccore) as references for blastn filtering (Table 2). A total of 20 sample libraries contained at least 10 HPgV-matching reads and showed a median of 8,185 reads (IQR 5207–15,863), equating 17,000 to 19 million total sequenced bases (Table 3). Near-complete genome sequences could be assembled from 17 of the libraries, and reduced genome coverage obtained from the 3 samples with sequence read totals <100,000. The sequence characteristics of the HPgV sequences were typical for members of this virus group: moderate sequence divergence between each other and to the HPgV reference sequence (9%–13%), extremely low dN/dS ratios (0.022–0.049) and evidence for extensive internal RNA secondary structure (mean folding energy differences of 10.5%–13.2%; Table 3). Persons infected with HPgV originating from the UK were infected with genotype 2, and those from South Africa harbored genotypes 1 and 5 (Figure 1; *18*). The exception was patient 89860282, a UK resident man enrolled in the Short Pulse Antiretroviral Therapy at seroConversion trial who was infected with a candidate novel genotype pegivirus in addition to a clade B HIV-1.

In addition to being detected at a lower frequency than HPgV in groups that listed sexual contact and injected drug use as potential risk factors for infection (Table 2), read totals for HHpgV-1 were >2 SDs below mean totals for HPgV. This finding is consistent with a lower degree of viremia.

### Validation of PCR for HHpgV-1

We compared sequences of the 2 HHpgV-1 samples with AK-790 and other group 2 pegiviruses (*6*) to identify conserved regions that might serve as binding sites for primers suitable for HHpgV-1 screening. A PCR based on primers hybridizing to a conserved region of group 2 pegiviruses (Table 1; *6*) and those previously described (AK1/AK2) were validated by using the original samples identified as positive on bioinformatic screening, the suspected false-positive sample (D1212), and a selection of samples in which HHpgV-1 sequences were not detected (Table 4). To cross-validate the assay for HPgV detection, primers were designed based on regions conserved in the NS3 region of group 1 pegiviruses (human, primate, and bat pegiviruses; Table 1).

**Table 4:** Validation of human pegivirus detection in plasma samples by using PCR*

No. samples	Metagenomic screen results†			Primers, no. positive		
HHpgV-1	HPgV	Group 2	ak1/ak2‡	Group 1
2	+	–		2	2	0
1	+/–	–		0	0	0
10	–	+		0	0	10
10	–	–		0	0	0
*HPgV, human pegivirus; HHpgV, human hepegivirus; +, positive; ­–, negative. †Virus status as determined by bioinformatic screening. ‡Nonstructural 3 genes as previously described (*6*).						

For groups 1 and 2 primers, PCR detection showed high concordance with bioinformatic screening (Table 4). By using group 2 primers, both samples that contained high numbers of HHpgV-1 reads on bioinformatic screening tested positive and the suspected negative sample (D1212) with only 3 reads was negative, as were the 20 controls in libraries that contained no HHpgV-1 sequences. Similar concordance between HPgV detection by PCR with library screening was observed in parallel (both methods identified 10 positive samples and 13 negative). The observed concordance between PCR- and bioinformatic-based screening methods for both virus groups validates both approaches for the wider screening for both virus groups in epidemiologic analyses.

### HHpgV-1 Detection in Case-Patients Transfused Multiple Times

We used pegivirus groups 1 and 2 PCRs to screen plasma samples from persons with hemophilia exposed to non–virally inactivated factor VII/IX concentrates and non–parenterally exposed controls (Table 2). Persons with hemophilia showed increased frequencies of HPgV viremia when compared with controls (18 of 195 compared with 1 of 50, respectively; Table 2), although this difference did not achieve statistical significance (p≈0.069 by Fisher exact test). One sample from a person with hemophilia was positive for HHpgV-1 by using group 2 primers; the amplicon sequence was 2.4%–4.8% divergent from AK-790, D1255, and D1220 between positions 4498–4896 in the AK-790 genome, with substitutions predominantly at synonymous sites. The sequence was phylogenetically distinct from the larger dataset of HHpgV-1 sequenced in the NS3 region (Figure 2; *17*).

## Discussion

This study used a combined approach of bioinformatic screening of metagenomic sequence libraries and pegivirus group-specific PCRs to investigate the frequency and risk group associations of HHpgV-1 infections. The cross-validation of these 2 screening methods provides reassurance that the methods used for detection of the 2 pegivirus groups were both sensitive and specific, notwithstanding the relatively infrequent detection of HHpgV-1 in the study populations.

The 2 different screening approaches clearly have their own advantages and disadvantages. Bioinformatic screening methods are able to detect a much broader range of genetic variants of a target virus that would require separate PCRs for their detection. As an example, HHpgV-1 was originally detected by bioinformatic screening of metagenomic libraries by using HPgV as the reference sequence (*6*), but the design of primers capable of detecting all pegiviruses is problematic and in practice may require separate assays for group 1 and 2 variants as used in this study. Metagenomic virtual screening could be easily extended by the use of multiple reference sequences representing a much wider range of viruses than would be practical for PCR, for which multiple assays would have to be developed, validated, and applied in complex, multiplexed formats. Library screening for human pathogens has been proposed as alternative to multiplex PCR for this purpose (*7*–*9*). Another advantage of bioinformatic screening is that it is usually possible to assemble near-complete genome sequences of the viruses being screened, which provides invaluable information for studies of its molecular epidemiology, transmission, and evolution. Both HHPgV-1 and 17 of the 20 HPgV variants detected by bioinformatic screening could be assembled in near-complete genome sequences (Table 3). The HPgV sequences represent a substantial increase on the number of complete genome sequences obtained to date and have identified further examples of rarely reported genotype 1 and 5 sequences, along with a putative new HPgV type (sample 89860282). In contrast, PCR amplicons are generally short and far less informative for strain identification or phylogenetic analysis, particularly if derived from highly conserved regions of the genome, as was the case for the HHpgV-1 and HPgV PCRs used in this study.

The specificity and sensitivity of bioinformatic screening is critically dependent on library quality; sequences derived from plasma samples or other largely acellular samples (cerebrospinal fluid, nasopharyngeal aspirates, urine) vary considerably in the numbers of contaminating host genomic sequences that may influence the effectiveness of screening for viral sequences in an unpredictable way (*19*). As demonstrated in this study, metagenomic libraries may be variably affected by contaminating sequences originating from other samples in the sequencing run, or may be bioinformatically contaminated from errors in reading identification tags for multiplexed sequencing reactions (*20*). In contrast, PCR-based screenings are capable of single copy target sensitivity in a wide range of sample types, and appropriate laboratory and assay design can entirely avoid false-positive results arising from sample or reagent contamination (*21*).

In this study, results from the 2 detection approaches for HHpgV-1 viremia were consistent and demonstrated the rarity of viremia with this virus in groups most at risk for parenterally and sexually transmitted virus infections. For example, only 1 person in the HGDS cohort showed viremia for HHpgV-1, despite previous extensive treatment with nonvirally inactivated factor VIII or IX concentrates (*10*). Exposure to bloodborne pathogens is attested by their universal seropositivity for HCV and high rate of HIV-1 infection (50% in those selected for this study). Despite the evidence for parenteral transmission of HHpgV-1 in the original study (*6*), our finding of a low frequency of detectable infection in this risk group is consistent with the originally reported low rate of viremia among persons with hemophilia (2/106 [*6*]). HHpgV-1 was similarly detected at low frequency in HCV-positive persons who inject drugs (2/120; Table 2), although persons in this risk group were almost universally infected with HCV from needle sharing.

In interpreting these results, we can rule out a poor sequence library or physical sample quality as the cause for nondetection of HHpgV-1. Viremia frequencies of the other pegivirus, HPgV, were comparable to those previously described in these risk groups, with elevated frequencies in those with histories of sexual exposure (8% in the HIV-positive persons in this study) previously reported to have high rates of HPgV active infection (*22*–*25*). Elevated frequencies are similarly reported in persons who inject drugs (*26*,*27*), which is consistent with the increased detection frequencies in this study (11%).

The findings of relatively low frequencies of HHpgV-1 viremia in the groups screened in this study suggest that it circulates less in human populations than HPgV, HCV, or HIV-1 or that infections are associated with a higher rate of clearance than for these other bloodborne viruses. Persistence over months or years was observed in 2 persons with hemophilia in the original study, although both blood recipients infected with HHpgV-1 cleared viremia within 241 and 281 days posttransfusion (*6*). The propensity of HHpgV-1 to persist for long periods, at least in some persons, is shared with many hepaciviruses and pegiviruses. Analysis of the coding regions of the 2 HHpgV-1 variants detected in this study revealed evidence for large-scale RNA secondary structure (mean folding energy differences of 7.6% and 8.1%), similar to that of the originally described HHpgV-1 sequence (*6*). This finding is within the range of values previously associated with host persistence in a wide range of positive-stranded mammalian RNA viruses, including HCV and HPgV in humans (*16*,*28*). Nevertheless, given the extensive exposure of the HGDS cohort investigated in this study to bloodborne viruses, the absence of detectable HHpgV-1 viremia in all but 1 of the persons in this study is more consistent with a higher rate of virus clearance for HCV and HPgV in this group rather than a lack of exposure.

Our findings potentially mirror those of our previous investigation of the parenterally transmitted parvovirus PARV4 in the HGDS cohort (*29*), where exposure is also closely associated with HCV and HIV through shared routes of transmission (*30*). Although all 195 study subjects were PCR-negative for PARV4 DNA at the end of the study period, 44% were seropositive for anti-PARV4 antibodies, and a process of acute infection followed by clearance was documented for a large number from whom serial samples were available over the period of infection. Without a serology assay for HHpgV-1, it is problematic to determine whether the lack of viremia detection in the HGDS cohort and other groups arose though lack of exposure or high rates of clearance of viremia.

In summary, this study used 2 complementary and cross-validated screening approaches to document frequencies of active infection of several different study groups with HHpgV-1 compared with other parenterally and sexually transmitted flaviviruses. Complementary screening of these risk groups for past exposure by using serology assays is required to understand more about its epidemiology, transmission routes, and host interactions.

## References

[1] Lipkin WI, Anthony SJ. Virus hunting. Virology. 2015;479–480:194–9. 10.1016/j.virol.2015.02.006 25731958

[2] Chiu CY. Viral pathogen discovery. Curr Opin Microbiol. 2013;16:468–78. 10.1016/j.mib.2013.05.001 23725672 PMC5964995

[3] Lei XY, Liu MM, Yu XJ. Severe fever with thrombocytopenia syndrome and its pathogen SFTSV. Microbes Infect. 2015;17:149–54. 10.1016/j.micinf.2014.12.002 25498868

[4] McMullan LK, Folk SM, Kelly AJ, MacNeil A, Goldsmith CS, Metcalfe MG, A new phlebovirus associated with severe febrile illness in Missouri. N Engl J Med. 2012;367:834–41. 10.1056/NEJMoa1203378 22931317

[5] Lipkin WI, Firth C. Viral surveillance and discovery. Curr Opin Virol. 2013;3:199–204. 10.1016/j.coviro.2013.03.010 PMC4310698 23602435

[6] Kapoor A, Kumar A, Simmonds P, Bhuva N, Singh CL, Lee B, Virome Analysis of transfusion recipients reveals a novel human virus that shares genomic features with hepaciviruses and pegiviruses. MBio. 2015;6:e01466–15 . 10.1128/mBio.01466-15 26396247 PMC4600124

[7] Petty TJ, Cordey S, Padioleau I, Docquier M, Turin L, Preynat-Seauve O, Comprehensive human virus screening using high-throughput sequencing with a user-friendly representation of bioinformatics analysis: a pilot study. J Clin Microbiol. 2014;52:3351–61. 10.1128/JCM.01389-14 25009045 PMC4313162

[8] Li L, Deng X, Mee ET, Collot-Teixeira S, Anderson R, Schepelmann S, Comparing viral metagenomics methods using a highly multiplexed human viral pathogens reagent. J Virol Methods. 2015;213:139–46. 10.1016/j.jviromet.2014.12.002 25497414 PMC4344864

[9] Greninger AL, Naccache SN, Federman S, Yu G, Mbala P, Bres V, Rapid metagenomic identification of viral pathogens in clinical samples by real-time nanopore sequencing analysis. Genome Med. 2015;7:99;0220–9. 10.1186/s13073-015-0220-9 PMC4587849 26416663

[10] Hilgartner MW, Donfield SM, Willoughby A, Contant CF Jr, Evatt BL, Gomperts ED, Hemophilia growth and development study. Design, methods, and entry data. Am J Pediatr Hematol Oncol. 1993;15:208–18. 10.1097/00043426-199305000-00009 8498644

[11] SPARTAC Trial Investigators. Fidler S, Porter K, Ewings F, Frater J, Ramjee G, Cooper D, et al. Short-course antiretroviral therapy in primary HIV infection. N Engl J Med. 2013;368:207–17. 10.1056/NEJMoa1110039 PMC4131004 23323897

[12] Matthews PC, Adland E, Listgarten J, Leslie A, Mkhwanazi N, Carlson JM, HLA-A*7401-mediated control of HIV viremia is independent of its linkage disequilibrium with HLA-B*5703. J Immunol. 2011;186:5675–86. 10.4049/jimmunol.1003711 21498667 PMC3738002

[13] Iles JC, Abby Harrison GL, Lyons S, Djoko CF, Tamoufe U, Lebreton M, Hepatitis C virus infections in the Democratic Republic of Congo exhibit a cohort effect. Infect Genet Evol. 2013;19:386–94. 10.1016/j.meegid.2013.01.021 23419346

[14] Yang X, Charlebois P, Gnerre S, Coole MG, Lennon NJ, Levin JZ, De novo assembly of highly diverse viral populations. BMC Genomics. 2012;13:475. 10.1186/1471-2164-13-475 22974120 PMC3469330

[15] Simmonds P. SSE: a nucleotide and amino acid sequence analysis platform. BMC Res Notes. 2012;5:50. 10.1186/1756-0500-5-50 22264264 PMC3292810

[16] Tamura K, Stecher G, Peterson D, Filipski A, Kumar S. MEGA6: Molecular Evolutionary Genetics Analysis version 6.0. Mol Biol Evol. 2013;30:2725–9. 10.1093/molbev/mst197 24132122 PMC3840312

[17] Simmonds P, Tuplin A, Evans DJ. Detection of genome-scale ordered RNA structure (GORS) in genomes of positive-stranded RNA viruses: Implications for virus evolution and host persistence. RNA. 2004;10:1337–51. 10.1261/rna.7640104 15273323 PMC1370621

[18] Tavaré S. Some probabilistic and statistical problems in the analysis of DNA sequences. In: Miura RM, editor. Lectures on mathematics in the life sciences. Volume 17. Providence (RI): American Mathematical Society; 1986. p. 57–86.

[19] Van Vliet KE, Muir P, Echevarria JM, Klapper PE, Cleator GM, Van Loon AM. Multicenter proficiency testing of nucleic acid amplification methods for the detection of enteroviruses. J Clin Microbiol. 2001;39:3390–2. 10.1128/JCM.39.9.3390-3392.2001 11526187 PMC88355

[20] Kircher M, Sawyer S, Meyer M. Double indexing overcomes inaccuracies in multiplex sequencing on the Illumina platform. Nucleic Acids Res. 2012;40:e3. 10.1093/nar/gkr771 22021376 PMC3245947

[21] Kwok S, Higuchi R. Avoiding false positives with PCR. Nature. 1989;339:237–8. 10.1038/339237a0 2716852

[22] Scallan MF, Clutterbuck D, Jarvis LM, Scott GR, Simmonds P. Sexual transmission of GB virus-C/hepatitis G virus. J Med Virol. 1998;55:203–8. 10.1002/(SICI)1096-9071(199807)55:3<203::AID-JMV4>3.0.CO;2-5 9624607

[23] Wu JC, Sheng WY, Huang YH, Hwang SJ, Lee SD. Prevalence and risk factor analysis of GBV-C/HGV infection in prostitutes. J Med Virol. 1997;52:83–5. 10.1002/(SICI)1096-9071(199705)52:1<83::AID-JMV13>3.0.CO;2-1 9131462

[24] Rubio A, Rey C, Sanchez Quijano A, Leal M, Pineda JA, Lissen E, Is hepatitis G virus transmitted sexually? JAMA. 1997;277:532–3. 10.1001/jama.1997.03540310030026 9032156

[25] Nerurkar VR, Chua PK, Hoffmann PR, Dashwood WM, Shikuma CM, Yanagihara R. High prevalence of GB virus C hepatitis G virus infection among homosexual men infected with human immunodeficiency virus type 1: Evidence for sexual transmission. J Med Virol. 1998;56:123–7. 10.1002/(SICI)1096-9071(199810)56:2<123::AID-JMV4>3.0.CO;2-A 9746067

[26] Dawson GJ, Schlauder GG. PilotMatias TJ, Thiele D, Leary TP, Murphy P et al. Prevalence studies of GB virus-C infection using reverse transcriptase polymerase chain reaction. J Med Virol. 1996;50:97–103. 10.1002/(SICI)1096-9071(199609)50:1<97::AID-JMV16>3.0.CO;2-V 8890047

[27] Liu HF, Goderniaux E, Burtonboy G, Goubau P. Molecular analysis of GB virus C/hepatitis G virus in HIV-1–positive intravenous drug users in Belgium. J Hum Virol. 1999;2:115–20 . 10225213

[28] Davis M, Sagan S, Pezacki J, Evans DJ, Simmonds P. Bioinformatic and physical characterisation of genome-scale ordered RNA structure (GORS) in mammalian RNA viruses. J Virol. 2008;82:11824–36. 10.1128/JVI.01078-08 18799591 PMC2583674

[29] Sharp CP, Lail A, Donfield S, Gomperts ED, Simmonds P. Virologic and clinical features of primary infection with human parvovirus 4 in subjects with hemophilia: frequent transmission by virally inactivated clotting factor concentrates. Transfusion. 2012;52:1482–9. 10.1111/j.1537-2995.2011.03420.x 22043925

[30] Matthews PC, Malik A, Simmons R, Sharp C, Simmonds P, Klenerman P. PARV4: an emerging tetraparvovirus. PLoS Pathog. 2014;10:e1004036. 10.1371/journal.ppat.1004036 24789326 PMC4006886

